# Sediment drying-rewetting cycles enhance greenhouse gas emissions, nutrient and trace element release, and promote water cytogenotoxicity

**DOI:** 10.1371/journal.pone.0231082

**Published:** 2020-04-02

**Authors:** José R. Paranaíba, Gabrielle Quadra, Iollanda I. P. Josué, Rafael M. Almeida, Raquel Mendonça, Simone Jaqueline Cardoso, Júlio Silva, Sarian Kosten, José Marcello Campos, Joseane Almeida, Rafael Lethournon Araújo, Fábio Roland, Nathan Barros

**Affiliations:** 1 Laboratório de Ecologia Aquática, Programa de Pós-Graduação em Ecologia, Universidade Federal de Juiz de Fora, Juiz de Fora, Brazil; 2 Laboratório de Limnologia, Programa de Pós-Graduação em Ecologia, Universidade Federal do Rio de Janeiro, Rio de Janeiro, Brazil; 3 Department of Ecology and Evolutionary Biology, Cornell University, Ithaca, NY, United States of America; 4 Grupo Baccan de Química Analítica, Departamento de Química, Universidade Federal de Juiz de Fora, Juiz de Fora, Brazil; 5 Departamento de Engenharia Metalúrgica e de Minas, Instituto Nacional de Ciência e Tecnologia (INCT) Acqua, Escola de Engenharia, Universidade Federal de Minas Gerais, Belo Horizonte, Brazil; 6 Department of Aquatic Ecology and Environmental Biology, Institute for Water and Wetland Research, Radboud University, Nijmegen, Netherlands; 7 Laboratório de Genética e Biotecnologia, Universidade Federal de Juiz de Fora, Juiz de Fora, Brazil; University of Western Australia, AUSTRALIA

## Abstract

Increased periods of prolonged droughts followed by severe precipitation events are expected throughout South America due to climate change. Freshwater sediments are especially sensitive to these changing climate conditions. The increased oscillation of water levels in aquatic ecosystems causes enhanced cycles of sediment drying and rewetting. Here we experimentally evaluate the effects of induced drought followed by a rewetting event on the release of carbon dioxide (CO_2_), methane (CH_4_), nutrients (nitrogen and phosphorus), and trace elements (iron, manganese, and zinc) from the sediment of a tropical reservoir in southeastern Brazil. Furthermore, we used bulb onions (*Allium cepa*) to assess the potential cytogenotoxicity of the water overlying sediments after rewetting. We found peaks in CO_2_ and CH_4_ emissions when sediments first transitioned from wet to dry, with fluxes declining as sediments dried out. CO_2_ emissions peaked again upon rewetting, whereas CH_4_ emissions remained unaltered. Our experiment also revealed average increases by up to a factor of ~5000 in the release rates of nutrients and trace elements in water overlying sediments after rewetting. These increased release rates of potentially toxic compounds likely explain the lower replication of *Allium cepa* cells (up to 22% reduction) exposed to water overlying sediments after rewetting. Our findings suggest that increased events of drought followed by rewetting may lead to a range of changes in freshwater ecosystems, including nutrient enrichment, increased toxicity following resuspension of contaminants, and higher emission of greenhouse gases to the atmosphere.

## Introduction

Global temperature changes can alter the circulation patterns of water and air masses, which have direct effects on precipitation regimes worldwide [1,2]. Accordingly, climate change projections suggest an increased occurrence of prolonged droughts followed by extreme precipitation events in different regions of South America [1,2]. Indeed, Brazil has experiencing years of extreme precipitation interspersed by years with abnormal droughts, including the incidence of unusually dry summers in typically rainy regions of the country’s Southeast [3].

Changes in the global climate patterns have been causing drastic modifications to various ecosystems on Earth [4]. Freshwater ecosystems such as rivers, lakes, wetlands, and reservoirs are particularly vulnerable to environmental changes. In addition, these ecosystems have been deeply undermined by human activities, including land-use changes, landscape fragmentation, river damming, and wastewater loading [5–7]. Damming, in particular, represents one of the most serious anthropogenic pressures on the world’s rivers over the last decades [5,8,9], and dam construction is expected to continue on the rise [10]. One important consequence of damming is the increased entrapment of terrestrially-derived elements into the sediment (e.g. organic and inorganic carbon, nutrients, trace elements) and the subsequent reduction of their transport to downstream ecosystems, including the ocean [5,11–13]. Another important consequence of damming is the intensification of microbial activity (e.g. decomposition, photosynthesis) within the constructed reservoir due to the transformation of a lotic system into a lentic one, leading to direct and indirect effects of element processing in the reservoir's sediment and water column [5,14].

The sediment represents one of the most important pools for the accumulation, processing, and transfer of elements in aquatic systems [15]. Elements may accumulate in the different geochemical layers of the sediment mainly through adsorption [16]. Thus, this sediment “memory” can provide snapshots of the aquatic ecosystems over time, recording information of environmental impact episodes and accumulating chemical species either used or produced by anthropogenic activities [17,18]. Sediment-trapped elements are not inert and may return to the water column through different pathways, such as resuspension events caused by wind or wave actions, dredging, bioturbation, biological transformations (e.g. photosynthesis, transport through the food chain), changes in the physical chemical properties of the sediment-water interface (e.g. redox, pH), and diffusion [15,19,20].

The incidence of extreme droughts decreases reservoir water levels, exposing large areas of previously submerged aquatic sediment to direct contact with the atmosphere. Exposed aquatic sediments have been reported as significant sources of greenhouse gases (GHG) to the atmosphere, especially carbon dioxide (CO_2_) and methane (CH_4_) [21–24]. However, when precipitation levels return to normal and the reservoir water level rises, these exposed sediments become submerged again. This rewetting phenomenon may favor the release of the remaining elements in the sediment to the water column due to the physical, chemical, and biological processes mentioned above. Although this is a natural process occurring on a seasonal scale, increased frequency of sediment drying and rewetting may exacerbate the release of elements buried in sediments either to the water column or directly to the atmosphere.

Enhanced release of elements and compounds from sediment to the water column may affect aquatic ecosystems. The release of nutrients such as nitrogen (N) and phosphorus (P) may support higher growth of bloom-forming phytoplankton, consequently increasing the internal production of organic matter and potentially leading to eutrophication [25,26]. Furthermore, exposure to trace elements (e.g. iron, manganese, zinc) may be toxic to various aquatic organisms and enhances biomagnification in the food chain [15,27,28]. The implications of exposing sediments/soils to the atmosphere and their subsequent rewetting have been described in the literature, particularly with respect to changes in GHG fluxes and nutrient release. In contrast, much less is known about contaminant remobilization (trace elements). In addition, little is known about the effects of such phenomenon in sediments from freshwater reservoirs worldwide.

Here, we address the potential environmental impacts caused by a cycle of drought-rewetting in the sediment of a tropical reservoir. The environmental impacts considered here were: (1) contribution to greenhouse effect, through the release of the GHGs CO_2_ and CH_4_; (2) contribution to eutrophication, due to the release of nutrients (nitrogen and phosphorus); and (3) contribution to toxicity, caused by the release of trace elements (iron, Fe; manganese, Mn; and zinc, Zn). Furthermore, we used a cytogenotoxicity test (Allium test) to compare possible cytogenotoxic effects of the overlying water from the induced-to-drought sediments (water samples from the rewetting period) with the overlying water from the sediments, which were kept permanently flooded. Our study was based on three hypotheses: (1) sediments under induced drying with subsequent rewetting show peaks of CO_2_ and CH_4_ emissions during both drying and rewetting periods, with total diffusive CO_2_ and CH_4_ fluxes being higher in the induced-to-drought sediments than in permanently flooded ones; (2) higher release rates and, therefore, higher total flux of nutrients and trace elements will be observed in the overlying water of the induced-to-drought sediments than in the permanently flooded ones; (3) cytogenotoxic effects will be observed in *Allium cepa* cells exposed to the overlying water from the rewetting period.

## Material and methods

### Sampling site characteristics

Chapéu D’Uvas reservoir (21° 33’S, 43° 35’W) is a 26-year-old reservoir constructed in Paraibuna River basin, 50 km downstream from the headwaters. It is located in the Atlantic forest biome [29] and has 12 km^2^ of maximum flooded area. Chapéu D’Uvas is mainly used for water supply purposes and is classified as oligotrophic (average of total nitrogen (TN) and total phosphorus (TP): 452 μg L^-1^ and 12 μg L^-1^, respectively) [30]. The region is characterized by dry winters and wet summers, with average annual temperature and precipitation ranging from 18 to 22 ºC and from 31 to 238 mm, respectively. The Federal University of Juiz de Fora holds a field station in the Chapéu D’Uvas reservoir (Núcleo de Integração Acadêmica para Sustentabilidade Sócioambiental, NIASSA); the field station is coordinated by two co-authors of this paper (F. Roland and N. Barros), who approved the field site access.

### Experimental setup

Eight sediment cores were sampled at a permanently flooded zone of the reservoir (depth ~25 m) using a gravity corer equipped with a hammer device (UWITEC, Mondsee, Austria). After removal, the cores were closed with plastic lids and transported to the laboratory. In the laboratory, the upper 20 cm of sediment from each core was transferred to polyvinyl chloride (PVC) incubation cores (inner diameter = 6 cm; height = 70 cm) and the overlying water of the sediment was carefully replaced by 25 cm of distilled water in order to exclude the substances dissolved in reservoir water.

Later, we randomly assigned 4 cores to be induced to drought with a subsequent rewetting event, whereas 4 cores were kept with constant water level. The experiment was performed in the absence of light to avoid primary production and to reduce external interferences to the biogeochemical processes under investigation. The cores were then acclimated over a controlled air temperature of 35°C (± 3°C). This temperature was chosen to enhance evaporation (drought simulation). Although 35°C is above naturally occurring temperature ranges for submerged sediments of Chapéu D’Uvas reservoir (~20–27°C), exposed sediments in drawdown areas may reach temperatures around 35°C in summer months (data not shown). Distilled water was daily added until the original level in both induced-to-drought and permanently flooded cores during the first week, and afterwards only in the permanently flooded cores. Distilled water (24 ± 2 mL of distilled water d^-1^—equivalent to 3% of the total overlaying water) was added to the cores one hour prior to the measurements. Air temperature and pressure were monitored using a portable weather station (Skymaster Speedtech SM-28, accuracy: 3%). Water temperature was monitored using a digital thermometer in skewer format (INCOTERM–resolution ± 0.5°C).

Following the experimental setup of Kosten et al. (2018) [21], we distinguished four different periods in our experiment that were described as: (i) “flooded period”, which corresponds to the period when we added distilled water daily to the induced-to-drought cores during the first week of the experiment plus the second week when we stopped adding distilled water and the overlying water, corresponding to the period that the sediments were inundated, started to evaporate (days 0–14); (ii) “drying period”, which started when the sediment from all induced-to-drought cores was directly exposed to the air, i.e. all overlying water had evaporated and the sediment moisture content started to decline (days 15–31); (iii) “dry period”, when the moisture content was considered as zero, i.e. the weight of the induced-to-drought cores ceased to decline (days 32–60); and (iv) “rewetting period”, when we started adding 100 mL of distilled water daily in the induced-to-drought cores until the end of the experiment (days 61–74). After starting the 100 mL additions in the rewetting period, water first filled the cracks created in the sediment in response to drying. It took two days until sediments became saturated and water began flooding over the surface of the sediment.

### Estimates of the diffusive fluxes of CO_2_ and CH_4_

After 24 h of acclimatization, we started to perform measurements of diffusive fluxes of CO_2_ and CH_4_ using an infrared Ultraportable Greenhouse Gas Analyzer (UGGA, Los Gatos Research Inc., detection limit of 1.5 x 10^−7^ mmol L^-1^ for CO_2_ and 2.76 x 10^−10^ mmol L^-1^ for CH_4_). Before starting the incubations, the UGGA was calibrated using known concentration standards for both CO_2_ and CH_4_. We used a gas-tight expandable PVC stopper equipped with O-rings sealer on the top of the stopper to record linear increments of CO_2_ and CH_4_ over 4 minutes in the headspace of the cores. Measurements with non-linear increments were caused by the emission of gas bubbles from the sediment and were therefore discarded. Then, further measurements were taken in order to capture only diffusive fluxes. Ebullitive fluxes were not included in our flux calculations due to human manipulation in the cores, which may favor the release of bubbles from the sediment to the water column and atmosphere, leading to interference in the flux results. Daily measurements were performed during the first and two last weeks of the experiment, whereas measurements were performed three times per week in the time in-between. All measurements were performed 1 h after adding distilled water in the permanently flooded cores throughout the experimental period, and in the induced-to-drought cores during the first week of the flooded period and during the rewetting period.

Total diffusive CO_2_ and CH_4_ emissions (as carbon (C) emissions) across the four experimental periods were estimated by calculating the area under the curve from the flux versus time plots. Moreover, total diffusive CH_4_ emissions were converted into CO_2_ equivalents (CO_2-eq_) by multiplying by a factor of 34, according to the CH_4_ Global Warming Potential (GWP) on a 100-year time horizon [1].

### Water sampling and replenishing

In order to understand how the release rate of compounds are affected during rewetting, we collected triplicate subsamples of 10 mL (i.e. 30 mL in total) of the overlying water at three different sampling times during the rewetting period (days 61, 67 and 74) for analysis of nutrient, trace elements, and cytogenotoxicity (described below). The subsamples were collected after the CO_2_ and CH_4_ flux measurements. At the end of each sampling day, 30 mL of distilled water was added to each core to compensate for the subsamples volume.

### Nitrogen and phosphorus analyses

To estimate the TN and TP release from sediment to the overlying water, the samples were analyzed by spectrophotometry (Beckman Coulter, DU640; wavelengths of 230 nm for TN and 885 nm for TP) after alkaline persulfate digestion for nitrogen [31] and potassium persulfate digestion for phosphorus [32]. Before analysis, glassware and volumetric materials were previously cleaned in an acid bath containing water and HNO_3_ (9:1, v/v) for 24 h and, then, washed with distilled water. The distilled water was tested for possible contamination; both TN and TP contents were below the limits of detection (5 μg L^-1^ for TN and 3.1 μg L^-1^ for TP).

TN and TP masses (mg) in each core were calculated by multiplying measured concentrations (mg m^-3^) by the amount of water in the core at the time of sampling (m^3^); total masses were adjusted to compensate for the amount of water collected for analysis in each day. TN and TP masses were then divided by the core area (0.0113 m^2^) to obtain masses per unit core area (mg m^-2^). We then regressed nutrient mass per unit area (mg of N or P m^-2^) against the sampling days (days 61, 67, and 74), with the slope of the relationship indicating TN and TP release rates over the sampling interval (mg of N or P m^-2^ d^-1^).

### Trace elements quantification

Trace elements quantification was performed using a Flame Atomization Atomic Absorption Spectrometer (FAAS), model (Thermo Scientific, Solaar M5 Series, Cambridge, United Kingdom). The wavelengths used were 248.3, 279.5, and 213.9 nm for Fe, Mn and Zn, respectively. The slit width was 0.2 nm for Fe, Mn, and Zn. The gas used was air/C_2_H_2_ at a flow rate of 1 L min^-1^ for Fe and Mn, and 1.2 L min^-1^ for Zn. Standard solutions were prepared from stock solutions (Quemis, Joinville, Brazil; 1000 mg L^-1^) and maintained in 0.1% of HNO_3_ (v/v). The concentrations used to construct the analytical curves ranged from 0.5 to 6 mg L^-1^ for Fe and Mn, and 0.25 to 3 mg L^-1^ for Zn. All glassware and volumetric materials were previously decontaminated in an acid bath containing water and HNO_3_ (9:1, v/v) for 24 h and then washed with deionized water. The distilled water used was also previously tested for possible contamination by trace elements and no contamination was observed (samples below limits of detection). The Limits of Detection (LD) was 0.011 mg L^-1^ for Fe and Mn, and 0.006 mg L^-1^ for Zn. The sensitivity, in terms of characteristics concentration, was 0.096, 0.042, and 0.017 mg L^-1^ for Fe, Mn, and Zn, respectively. We calculated the release rate of trace elements following the same approach described for TN and TP.

### Cytogenotoxicity test

The cytogenotoxicity test was performed using *Allium cepa* (onion) bulbs. First, the bulbs were placed in small cups with distilled water to stimulate root emergence. After 24 h, the bulbs were randomly exposed to permanently flooded and induced-to-drought water samples from the three different times (days 61, 67 and 74), in triplicates, and were kept in a Biological Oxygen Demand incubator (BOD) for 24 h. Afterward, the roots were washed and fixed in a cold solution of ethanol and acetic acid (3:1 v/v) for 24 h. Then, the roots were re-washed and slides were prepared using the squashing technique (for details, see Quadra et al. 2019 [33]). The slides were inspected at 400x magnification using an optical microscope to evaluate mitotic index and chromosomal aberrations: (i) aneugenic aberrations, which are usually related to chromosome losses, less adherence, and multipolarity; and (ii) clastogenic aberrations, which are related to DNA breaks [33,34].

### Statistical procedures

We used an unpaired *t-test* to assess differences in the diffusive flux of CO_2_ and CH_4_ between induced-to-drought and permanently flooded cores within each experimental period (flooded, drying, dry and rewetting). The unpaired *t-test* was also used to assess the differences between induced-to-drought and permanently flooded cores in relation to the releases of nutrients that supposedly occurred after rewetting. The same statistical procedure was adopted to the cytogenotoxic analysis. We log-transformed diffusive CO_2_ data in order to meet the assumption of normality and homoscedasticity. Cubic root function was applied for diffusive CH_4_ data. Nutrient (TN and TP) and cytogenotoxicity data met normality assumptions and, therefore, no transformation was required. Trace element data did not meet the assumptions of normality and homoscedasticity, and the non-parametric Wilcoxon test was adopted to test the differences between groups. Cohen’s *d* test was applied to determine the effect size of the CO_2_ and CH_4_, nutrients, trace elements, and cytogenotoxicity data. We assumed *p* < 0.05 as the threshold level of acceptance for all statistical tests. Statistical analyses were performed using JMP (version 14.0.0).

## Results

### Diffusive fluxes of CO_2_ and CH_4_ during a drying-rewetting cycle

**Fig 1:**
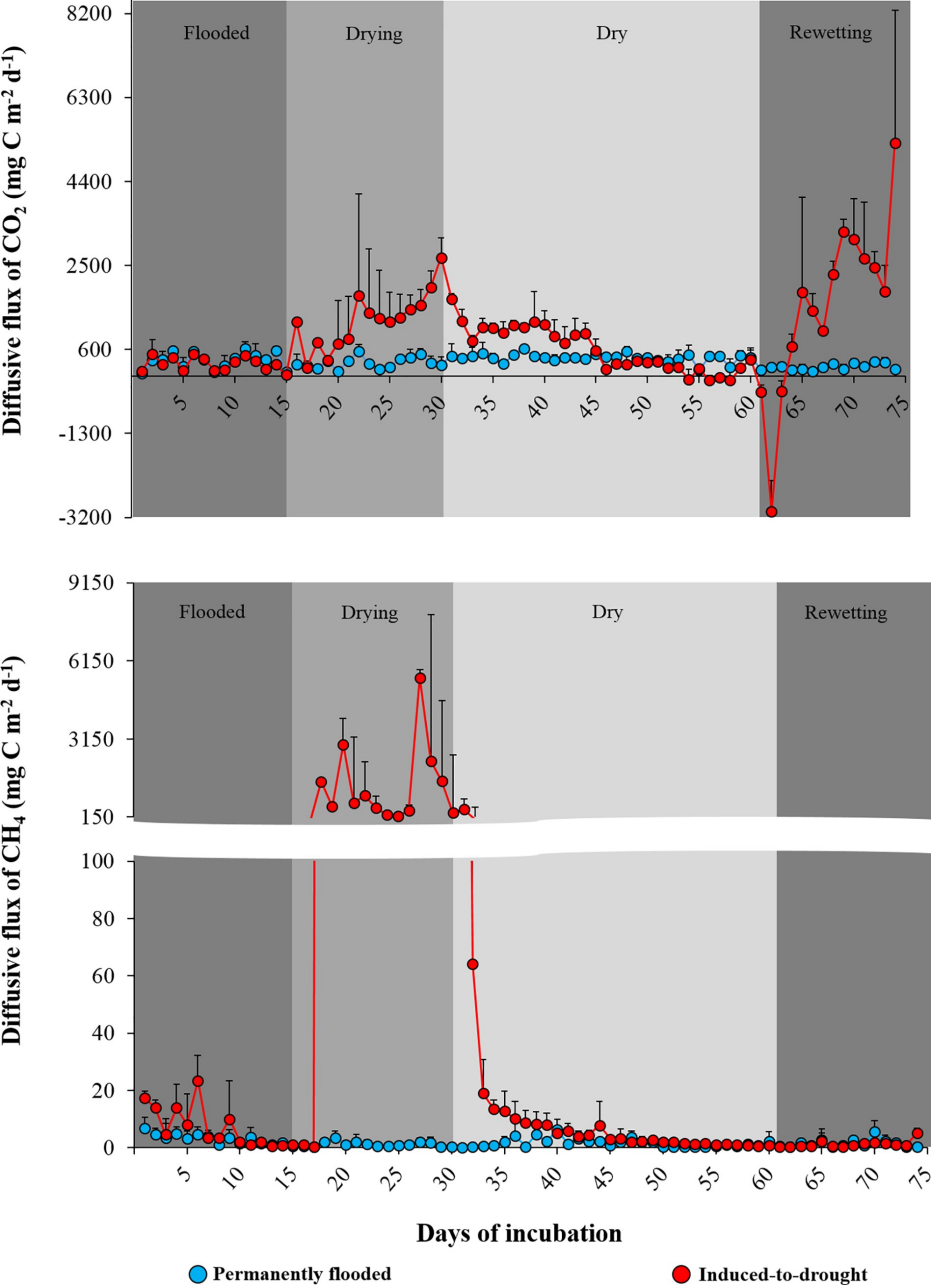
**Diffusive flux of CO**_**2**_ **(upper) and CH**_**4**_ **(bottom) (average ± standard deviation; mg C m**^**-2**^ **d**^**-1**^**) from permanently flooded (blue) and induced-to-drought cores (red) across the different experimental periods.** For a better view of results, only the upper standard deviation is shown.

We observed large variability in diffusive fluxes of CO_2_ and CH_4_ from induced-to-drought cores compared to the permanently flooded cores along the experimental periods (Fig 1, Table 1). The transition from the flooded to the drying period caused a peak in diffusive emissions for both gases (Fig 1), with values reaching up to 6677 mg C m^-2^ d^-1^ for CO_2_ and 13696 mg C m^-2^ d^-1^ for CH_4_ in the induced-to-drought cores. As sediments dried out, diffusive emissions of both gases declined to values comparable to those observed in the initial flooded period (Fig 1). Rewetting dried-out sediments resulted in a new peak in CO_2_ fluxes, but CH_4_ fluxes remained unaffected (Fig 1). Notably, rewetting led to the highest rates of CO_2_ emission throughout the experiment, with values reaching up to 9840 mg C m^-2^ d^-1^.

**Table 1:** Average ± standard deviation, median (bold), range (between parenthesis) of the diffusive CO_2_ and CH_4_ fluxes (mg C m^-2^ d^-1^), as well as total diffusive carbon emissions (mg C m^-2^ and g CO_2-eq_ m^-2^) from the permanently flooded and induced-to-drought cores across the four experimental periods (flooded, drying, dry and rewetting). Statistical results from the unpaired *t-test* are shown as *t-Ratio* and *p* values (between parenthesis) for each experimental period. Results from Cohen’s d Effect Size Test (*ES*) are shown right after *t-test* results.

			Diffusive CO_2_ emissions					Diffusive CH_4_ emissions					Total emission as CO_2-eq_ (g CO_2-eq_ m^-2^) ^*a*^	
	Flux (mg C m^-2^ d^-1^)				Total emission (mg C m^-2^)		Flux (mg C m^-2^ d^-1^)				Total emission (mg C m^-2^)			
	Permanently Flooded	Induced-to-drought	*t-Ratio (p)*	*ES*	Permanently Flooded	Induced-to-drought	Permanently Flooded	Induced-to-drought	*t-Ratio (p)*	*ES*	Permanently Flooded	Induced-to-drought	Permanently Flooded	Induced-to-drought
**Flooded period**	375 ± 170	293 ± 141	*1*.*35 (0*.*18)*	-*42*.*8–209*.*4*	5271 ± 111	4050 ± 92	3.2 ± 1.6	8.6 ± 7.8	*1*.*95 (0*.*073)*	0.13*–8*.*3*	42 ± 1.3	96 ± 5.4	6.7	7.4
** **	**374**	**298**					**3.4**	**5.7**						
** **	(55–619)	(108–496)					(0.9–6.8)	(0.5–23)						
**Drying period**	296 ± 131	1225 ± 667	*5*.*45 (0*.*0001)*	528.2–1276	5186 ± 98	21433 ± 559	1 ± 0.8	1065 ± 1376	*3*.*09 (0*.*007)*	363.3–1765	18 ± 0.7	18139 ± 1055	5.8	638
** **	**272**	**1302**					**0.8**	**512**						
** **	(87–562)	(30–2694)					(0.2–3.4)	(0.3–5510)						
**Dry period**	413 ± 95	566 ± 450	*1*.*75 (0*.*09)*	-21.9–326.9	11856 ± 68	15609 ± 441	1.7 ± 1.5	7 ± 12	*2*.*3 (0*.*03)*	0.6–9.6	48 ± 1	165 ± 8	13.5	21.2
	**414**	**374**					**1.3**	**3**						
** **	(134–627)	(-101–1244)					(0.2–6.2)	(0.5–64)						
**Rewetting period**	206 ± 67	1600 ± 1926	*2*.*6 (0*.*02)*	294.1–2492	2736 ± 51	19921 ± 1644	1.5 ± 1.4	1.3 ± 1.2	*0*.*5 (0*.*62)*	-0.08–1.3	20 ± 1	14 ± 0.7	3.4	20.4
** **	**198**	**1924**					**0.8**	**0.8**						
** **	(106–329)	(-3065–5291)					(0.2–5.6)	(0.3–5.1)						
**Total period**					25050 ± 111	61013 ± 924					128 ± 1.2	18415 ± 677	29.4	687
** **														
** **														

^a^ CH_4_ emissions were converted into CO_2-eq_ emission using a GWP of 34 on a 100-year time horizon (IPCC 2013).

Considering the entire length of the experiment, total diffusive CO_2_ and CH_4_ emissions (as C mass) from the induced-to-drought cores were, respectively, about 2.5 and 144 times higher than total diffusive CO_2_ and CH_4_ emissions observed in the permanently flooded cores (Table 1). In terms of CO_2-eq_, induced-to-drought cores emitted about 24 times more than the permanently flooded cores (Table 1).

No statistical differences were observed in both CO_2_ and CH_4_ fluxes between groups during the flooded period (Table 1). However, the diffusive flux of CO_2_ and CH_4_ significantly differed between induced-to-drought and permanently flooded cores during the drying period, during the dry period for CH_4_, and during the rewetting period for CO_2_ (Table 1).

### Nitrogen and phosphorus release during rewetting

**Fig 2:**
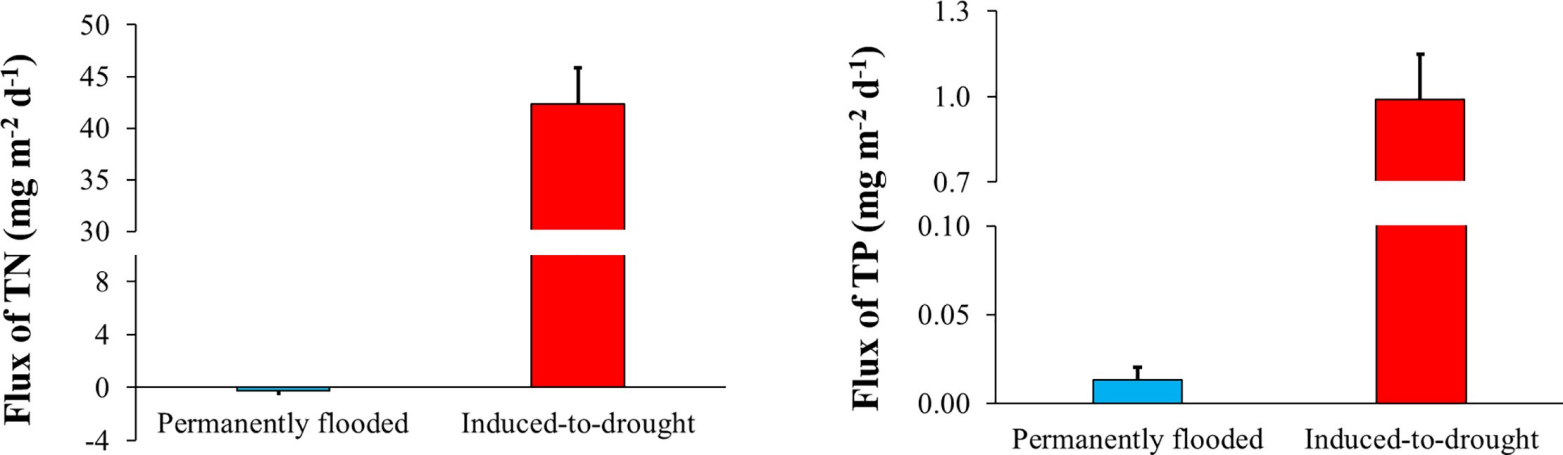
**Average ± standard error of total nitrogen (TN) and total phosphorus (TP) release rates (mg m**^**-2**^ **d**^**-1**^**) from permanently flooded (blue) and induced-to-drought cores (red)during the rewetting period.** For a better view of results, only the upper standard error is shown.

The release rate of TN ranged from -1.4 to 1.1 mg m^-2^ d^-1^ (average ± SD: -0.2 ± 1, median: -0.8 mg m^-2^ d^-1^) in the permanently flooded cores, and from 33.4 to 52.7 mg m^-2^ d^-1^ (average ± SD: 42.4 ± 7, median: 41.1 μg m^-2^ d^-1^) in the induced-to-drought cores (Table 2). The release rate of TP ranged from -0.007 to 0.03 mg m^-2^ d^-1^ (average ± SD: 0.01 ± 0.01, median: 0.008 mg m^-2^ d^-1^) in the permanently flooded cores, and from 0.6 to 1.4 mg m^-2^ d^-1^ (average ± SD: 1 ± 0.3, median: 0.7 mg m^-2^ d^-1^) in the induced-to-drought cores (Table 2). TN and TP fluxes to the overlying water of the induced-to-drought cores were consistently higher than those observed in the permanently flooded cores (Fig 2 and Table 2), with values up to ~380 times higher for nitrogen (*t-Ratio = 10*.*63*, *p = 0*.*001*) and ~210 times higher for phosphorus (*t-Ratio = 5*.*26*, *p = 0*.*01*). Results from Cohen’s *d* test revealed a confidence interval (lower 95%—higher 95%) ranging from 32.8 to 52.4 mg m^-2^ d^-1^ (*p = 0*.*001*) for TN, and from 0.5 to 1.4 mg m^-2^ d^-1^ (*p = 0*.*01*) for TP.

**Table 2:** Nutrient release rates (TN and TP—mg m^-2^ d^-1^) and r^2^ from the regression of nutrient mass per unit area against sampling interval for each core of the permanently flooded and induced-to-drought groups during the rewetting period. Statistical results from the unpaired *t-test* are shown as *t* and *p* values.

Nutrient	Group	Core number	Flux (mg m^-2^ d^-1^)	r^2^	Statistical significance
**Total Nitrogen**	Permanently flooded	C1	-0.8	0.789	*t = 10*.*63 p = 0*.*001*
		C2	1.1	0.977	
		C3	-1.4	0.696	
		C4	0.2	0.313	
**Total Nitrogen**	Induced-to-drought	T1	42.3	0.979	
		T2	52.7	0.986	
		T3	41.1	0.995	
		T4	33.4	0.954	
**Total Phosphorus**	Permanently flooded	C1	-0.01	0.093	*t = 5*.*26 p = 0*.*01*
		C2	0.03	0.344	
		C3	0.01	0.039	
		C4	0.02	0.038	
**Total Phosphorus**	Induced-to-drought	T1	1.4	0.999	
		T2	1.1	0.968	
		T3	0.7	0.971	
		T4	0.6	0.985	

### Trace element release during rewetting

**Fig 3:**
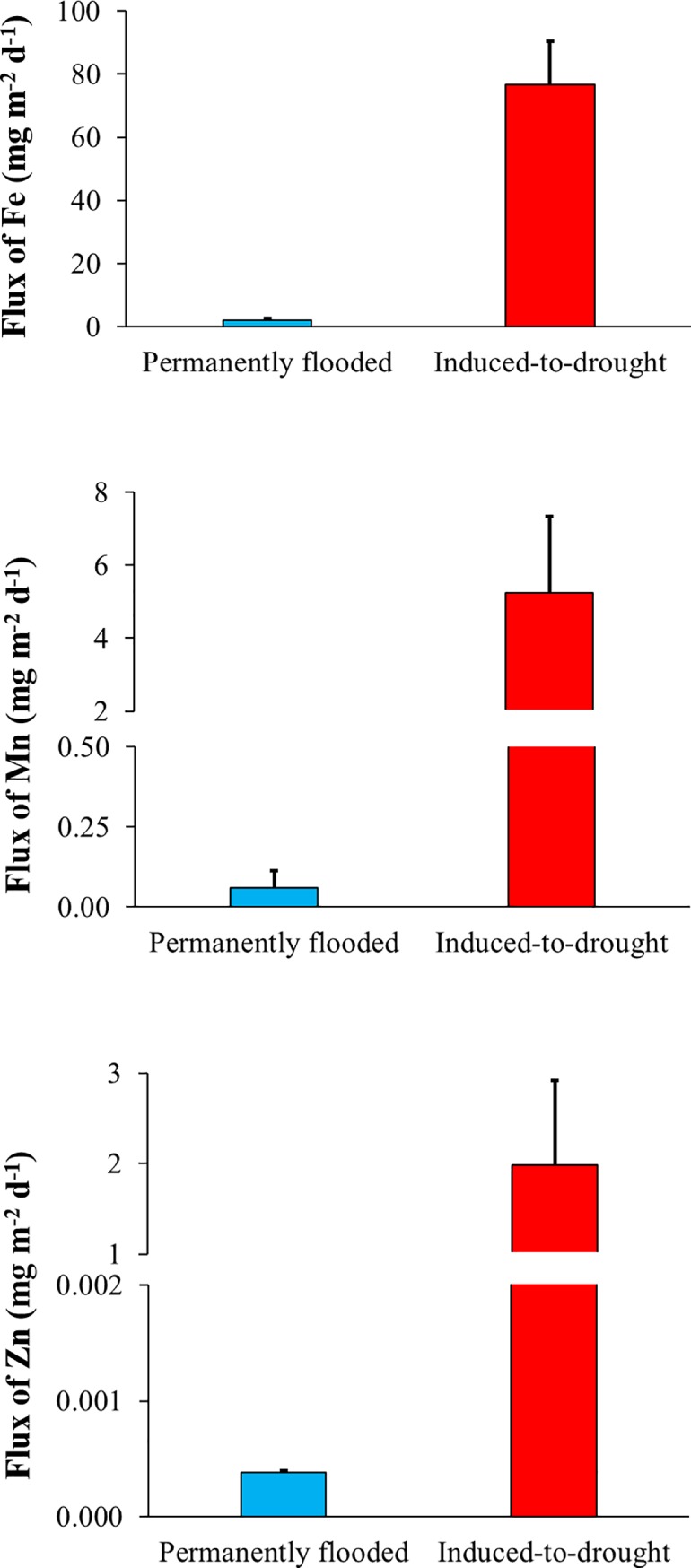
Average ± standard error of Iron (Fe), Manganese (Mn) and Zinc (Zn) release rates (mg m^-2^ d^-1^) from permanently flooded (blue) and induced-to-drought cores (red) during the rewetting period. For a better view of results, only the upper standard error is shown.

Statistically higher release rates and, consequently, higher total flux of Fe, Mn, and Zn were observed in the induced-to-drought cores compared to permanently flooded cores (Fe: *Z = 2*.*16*, *p = 0*.*03*; Mn: *Z = 2*.*21*, *p = 0*.*02*; Zn: *Z = 2*.*21*, *p = 0*.*02*) (Fig 3 and Table 3). Average release rates of dissolved Fe, Mn, and Zn observed in induced-to-drought cores were 40, 86, and 5220 times, respectively, higher than the average release rates observed in permanently flooded cores. Results from Cohen’s *d* test revealed a confidence interval ranging from 35.4 to 114 mg m^-2^ d^-1^ (*p = 0*.*03*) for Fe, from -0.7 to 11.1 mg m^-2^ d^-1^ (*p = 0*.*02*) for Mn, and from -0.6 to 4.6 mg m^-2^ d^-1^ (*p = 0*.*02*) for Zn.

**Table 3:** Trace element release rates (Fe, Mn, and Zn—mg m^-2^ d^-1^) and r^2^ from the regression of trace element mass per unit area against sampling interval for each core of the permanently flooded and induced-to-drought groups during the rewetting period. Statistical results from Wilcoxon test are shown as *Z* and *p* values.

Trace element	Group	Core number	Flux (mg m^-2^ d^-1^)	r^2^	Statistical significance
**Iron**	Permanently flooded	C1	3.81	0.980	*Z = 2*.*16 p = 0*.*03*
		C2	1.54	0.906	
		C3	1.14	0.999	
		C4	1.26	0.873	
**Iron**	Induced-to-drought	T1	111.03	0.986	
		T2	77.94	0.983	
		T3	83.94	0.943	
		T4	33.65	0.998	
**Manganese**	Permanently flooded	C1	0.24	0.788	*Z = 2*.*21 p = 0*.*02*
		C2	0.0007	0.711	
		C3	0.0007	0.711	
		C4	0.0007	0.711	
**Manganese**	Induced-to-drought	T1	1.66	0.782	
		T2	11.44	0.994	
		T3	6.71	0.895	
		T4	1.16	0.917	
**Zinc**	Permanently flooded	C1	0.0004	0.711	*Z = 2*.*21 p = 0*.*02*
		C2	0.0003	0.019	
		C3	0.0004	0.711	
		C4	0.0004	0.711	
**Zinc**	Induced-to-drought	T1	0.05	0.999	
		T2	4.56	0.791	
		T3	2.97	0.996	
		T4	0.34	0.839	

### Cytogenotoxic responses between groups

**Fig 4:**
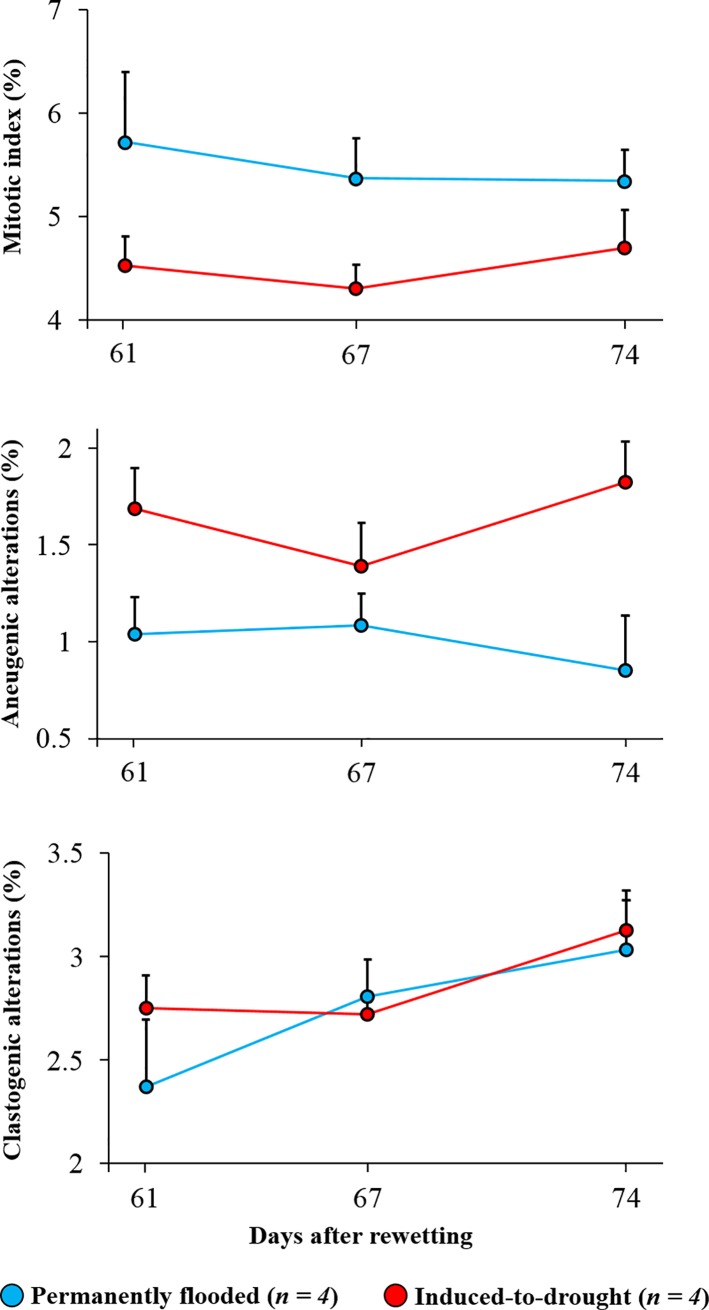
Average ± standard deviation of mitotic index, and aneugenic and clastogenic alterations from *Allium cepa* tests between groups (permanently flooded: blue, and induced-to-drought: red) on different sampling days during the rewetting period. For a better view of results, only the upper standard deviation is shown.

Significant mitotic index reduction was observed in *Allium cepa* cells exposed to water samples from induced-to-drought cores when compared to cells exposed to permanently flooded water samples (12 to 22% less cell replication; *t-Ratio = 5*.*26*, *p < 0*.*0001*) (Fig 4). Moreover, the aneugenic alterations were 28 to 114% higher in cells exposed to water samples from the induced-to-drought cores than the cells exposed to permanently flooded water samples (*t-Ratio = 5*.*79*, *p < 0*.*0001*) (Fig 4). No statistical difference was observed between groups in relation to clastogenic alterations (*t-Ratio = 0*.*95*, *p = 0*.*35*) (Fig 4). Results from Cohen’s *d* test revealed a confidence interval ranging from 0.59 to 1.35% (*p* < 0.0001) for mitotic index, from 0.41 to 0.87% (*p* < 0.0001) for aneugenic alterations, and from 0.15 to 0.4% (*p* = 0.35) for clastogenic alterations.

## Discussion

### Sediment drying with subsequent rewetting boosts GHG emission

We observed a significant increase of CO_2_ and CH_4_ emissions resulting from the drying and rewetting of freshwater sediments, similar to what has been found in previous studies [21,35–38]. The observed pattern is attributed to a phenomenon generally known as the "Birch effect", in reference to studies carried out by H.F. Birch in the 1950s and 1960s on the effects of droughts and rewetting events on the carbon and nitrogen cycle in agricultural and forest soils [39,40].

Accordingly, the biological processes that occur in aquatic sediments are directly associated with its water content [41,42]. According to our findings, the first peak of both CO_2_ and CH_4_ emissions occurs in sediments shortly after the sediment surface is exposed to the atmosphere (in our induced-to-drought cores these peaks corresponded to the first and second days after the overlying water was absent, Fig 1). Subsequently, during the entire drying period, the magnitude of CO_2_ and CH_4_ emissions from the induced-to-drought cores remained substantially higher than the emissions observed in the permanently flooded cores (4 and 1000 times higher for CO_2_ and CH_4_, respectively, after 15 days of drying) (Fig 1).

When sediments are exposed to direct contact with the atmosphere, we may expect a series of changes ultimately affecting the CO_2_ and CH_4_ dynamics, as follows: (i) the now exposed sediment has direct contact with atmospheric oxygen stimulating the decomposition of the usually high amount of labile organic matter in the surface sediment [43] and resulting in substantial carbon losses to the atmosphere [21]; (ii) as drought persists, sediment may crack causing the penetration of oxygen into deeper layers, which triggers more organic matter mineralization [35]; (iii) the cracks formed by the sediment desiccation may also favor the release of gases, that were retained in lower parts of the sediment, to the atmosphere, especially CH_4_, which tends to form gas bubbles (not accounted here) in the sediment due to its low solubility [21,44]; (iv) the gas-exchange velocity between the sediment-air interface is much faster (> 10000 times faster) than between the water-air interface, which considerably increases the diffusive carbon emissions between sediment-air interface [45]; (v) the boosted organic matter degradation by the increase in oxygen availability tends to decrease the pore water pH, potentially leading to gas dissolutions (i.e. CO_2_) from the sediment into the atmosphere [46].

When the water content of the sediments approached zero (i.e. when the weight of the induced-to-drought cores stabilized, ~30 days after the start of the incubations), we observed a substantial decrease in the sediment CO_2_ and CH_4_ emissions from the induced-to-drought cores (Fig 1). The paucity of water in previously submerged sediments likely promoted the desiccation of the sediment microbial communities [24,42,47], directly affecting their capacity to decompose organic matter into CO_2_ and CH_4_. We observed a slightly significant difference in CH_4_ emissions between the induced-to-drought and permanently flooded cores during the dry period (*t-Ratio = 2*.*3*, *p = 0*.*03*), caused by the high fluxes registered in the first days after the sediment was completely dry (Fig 1). It is possible that during this period there was still some microbial activity or that these fluxes correspond to the emission of the remnant gases produced during the drying phase.

Finally, the rewetting simulation resulted in a rapid, and short, influx of CO_2_ from the atmosphere to the recent overlying water of the induced-to-drought cores during the first three days of the rewetting period (Fig 1). The observed CO_2_ uptake may be caused by a possible lag-phase between rewetting and initiation of microbial production of CO_2_ leading to a flux of atmospheric CO_2_ into the potentially CO_2_-undersaturated distilled water. However, after 96 h of rewetting, the induced-to-drought cores became, again, strong CO_2_ sources to the atmosphere, reaching the highest recorded values until the end of the experiment (Fig 1). On the other hand, we did not observe any CH_4_ peaks occurring in the induced-to-drought cores during the rewetting period, with values similar to those observed in the permanently flooded cores (Fig 1). Some studies describe that 24 h of wet conditions are enough to stimulate heterotrophic microbial activities in the surface of sediments, promoting shifts in the microbial communities and increasing their biomass [35,48]. Borken et al. (2003) [37] and Kosten et al. (2018) [21] showed that rewetting events on sediments induced to drought triggered instantaneous CO_2_ emissions to the atmosphere, which remained increasing as the water content was also increasing. The same pattern was also observed in situ by Fromin et al. (2010) [35], who reported peaks of CO_2_ emissions after a rain event on dry sediments from a Mediterranean pond. Accordingly, the occurrence and magnitude of CO_2_ and CH_4_ emissions have been related to the frequency and intensity of dry and rewetting periods. For instance, different microbial responses (respiration) were observed in sediments that experienced distinct periods of dry conditions as well as the frequency of rewetting phases [21,35,49]. Therefore, the duration of droughts in conjunction with different rewetting periods may be considered a stressful process for microbial communities, playing a crucial role in the microbial dynamics submitted to such unstable conditions, resulting in contrasts of GHG production/emission [35,50].

Even though ebullition was observed at certain times throughout the experimental time in both groups, our findings do not take into account the contribution of this CH_4_ release pathway, which tends to increase in intensity when the water column lowers [51]. Moreover, possible incomplete carbon mineralization not resulting in CO_2_ or CH_4_ production but in dissolved organic carbon production, is not captured by our analyses. In addition, we may have also missed a CO_2_ sink by suppressing photosynthesis in our dark experimental conditions.

### Rewetting-related release of nitrogen and phosphorus

The rewetting of desiccated sediments led to substantial releases of nitrogen and phosphorus from the sediment to the overlying water in the induced-to-drought cores, where the release rates were higher than those observed in the permanently flooded cores throughout the rewetting period (14 days; Fig 2). When previously exposed sediments are re-flooded, an initial flush of nitrogen and phosphorus usually occurs to the overlying water (the so-called Birch effect) [39,52,53], mainly due to the enhancement of aerobic organic matter mineralization that tends to accumulate inorganic nitrogen in dry sediments, and due to the release of nitrogen and phosphorus bound to organisms that died when the sediments were drying out [35,41,54].

Previous studies have shown the release of phosphorus during drying-rewetting events [55,56], and the longer the drought period was, the greater subsequent release of phosphorus [56]. Moreover, events of drought in sediments increase the crystallinity of iron species, which leads to a loss of phosphorus binding capacity [56,57]. Our results also corroborate previous studies showing nitrogen release from sediments after drying and rewetting events, mostly in the form of N-ammonium [54,58,59]. N-ammonium can be toxic to aquatic fauna, such as fish and crustaceans [60], which means that the effects of drying and rewetting of sediments may increase the incidence of ammonium toxicity. In addition, our study site is oligotrophic, but if we investigate a nutrient-enriched aquatic ecosystem, it is likely that the amount of nitrogen and phosphorus released may be even higher [61]. Beyond the quantity of nutrients in the sediments, other factors may influence these nutrient releases, such as the history of drying and rewetting events, the exposure time to air during the dry period, sediment properties and microbial community metabolism [41,54,56,57,62].

After rewetting, microbial activity picks up, possibly in part due to the higher availability of nutrients as a result of drought induced cell lysis [41]. This enhanced metabolic activity quickly results in anaerobic conditions triggering denitrification and the release of iron-bound phosphorus [41]. Last but not least, rewetting exposed sediments may pose a risk (stress) for the sediment biota, which will potentially face cell lysis and, consequently, release more intracellular nitrogen and phosphorus to the overlying water [49,57].

The observed strong increase in sediment nutrient release as a result of desiccation-rewetting may favor eutrophication in our study system. In addition, our results substantiate that climate change related to changes in precipitation patterns may affect nutrient cycling in aquatic ecosystems globally [41].

### Rewetting-related release of trace elements

The contamination history of aquatic ecosystems may be a factor that influences the amount of trace elements released after the exposures of droughts and rewetting. The watershed of our study site, however, does not contain much urban or industrial development (land cover: ~66% of grassland, ~30% of natural forest, and ~4% of Eucalyptus plantation) [63] and, therefore, we did not expect high release rates of trace elements. On the other hand, high concentrations of Fe have already been reported in the sediments of Chapéu D’Uvas reservoir [27].

Our results showed that Fe, Mn and Zn release rates were, in general, higher in the induced-to-drought water samples than in the permanently flooded water samples (Fig 3). This means that an effect of drying and rewetting sediments was observed on the release of trace elements to the water column. The mobility of trace elements changes during drying and rewetting periods, possibly due to alterations in physical-chemical properties such as pH and redox potential [15,64,65]. When sediments are exposed to oxygen, a release of trace elements such as Fe and Zn is expected mainly due to changes in pH that consequently decrease the buffer capacity of the sediments [64]. Another possible explanation that corroborates the release of trace elements is their affinity for organic matter, which under oxidizing conditions may compromise the adsorption capacity of sediments, leading to the release of these elements to the water column after rewetting [66,67].

A strong release of Fe and Mn [59,68], as well as Al and Zn [68], have been found after rewetting events in exposed sediments. The Fe cycle is coupled to the phosphorus cycle [56], and the sediments exposed to oxygen may alter the production of iron species, causing changes in these biogeochemical cycles [69]. The release of contaminants in seasonal rivers was reported by Ademollo et al. (2011) [70], which showed that the bioavailable fraction of trace elements was more frequently found. This observation has ecological importance since the bioavailable fractions are more relevant in terms of environmental risks [27,70]. Thus, according to our results and previous studies, the maintenance of flooded sediments is important for the management of polluted areas.

The release rates of trace elements reported here may not likely lead to concentrations in the overlying water capable to cause acute and chronic toxicity on aquatic organisms, such as Mn in *Ceriodaphnia dubia* and *Hyalella Azteca* [71], Zn in *Litopenaeus vannamei* and *Rhithrogena hageni* [72,73], as well as for Fe in *Asellus aquaticus* and *Leptophlebia marginata* [74,75]. However, as above-mentioned, the release of compounds depends also on the contamination history of the aquatic ecosystem, where a more polluted environment may release more trace elements into the water column, likely leading to adverse effects on aquatic organisms. Beyond toxic effects, the trace elements may cause other environmental impacts, such as eutrophication. For example, Fe plays an important role in primary production and is, therefore, a limiting nutrient in aquatic ecosystems [76]. However, when in excess, it may lead to toxicity in aquatic organisms [75].

### Cytogenotoxic effects of compounds released upon rewetting

Allium test is a highly sensitive bioassay whose results correlates with those observed in other organisms; thus, it is widely used to investigate chemical contamination and toxicity effects in aquatic ecosystems [77,78]. The mitotic index indicates the number of cells in division during the cell cycle [34]. Allium cells exposed to induced-to-drought water samples showed a lower number of cells replicating when compared to cells exposed to permanently flooded water samples (Fig 4). Aneugenic effects are related to toxic effects in the cells and they are expressed as chromosomal alterations such as chromosome losses, delays, adherence, multipolarity and C-metaphases [34]. The aneugenic alterations were higher in the cells exposed to induced-to-drought water samples (Fig 4). Clastogenic effects are related to DNA breaks and they are expressed in chromosomal alterations such as bridges and breaks [34]. We did not observe significant difference in clastogenic effects between cells exposed to induced-to-drought and permanently flooded water samples (Fig 4). Given that only aneugenic alterations were observed, rather than both alterations studied (aneugenic and clastogenic), the contaminants presented in the water overlying sediments are likely related to the induction of chromosomal alterations linked to mitotic spindle dysfunctions [79,80].

Our results indicate the possibility of adverse effects occurring after sediment exposure followed by rewetting events. The cell proliferation was affected as well as toxic effects were observed, expressed by mitotic index and aneugenic effects. Previous studies reported cytogenotoxic effects of trace elements (Al, Cd, Fe, Mn, and Zn) using the Allium test [33,81,82]. Moreover, N-ammonium was also related to cause genotoxicity in *Oreochromis niloticus* [83]. Then, the cytogenotoxic results reported here may be attributed to synergistic effects of trace elements and nutrients (including other elements and compounds not investigated here) released during rewetting in the induced-to-drought sediments.

## Conclusions

This study showed different consequences of a cycle of induced drought followed by rewetting on the dynamics of GHG, nutrients, and trace elements in the sediment of a tropical reservoir. Our findings confirmed our three postulated hypotheses: (i) CO_2_ and CH_4_ emission peaks occurring at distinct experimental periods in induced-to-drought cores, with average fluxes up to 140 times higher than those observed in the permanently flooded cores; (ii) higher release rates of nutrients and trace elements in the overlying water of the induced-to-drought cores, reaching average values up to 206 times (TN) and 5220 times (Zn) higher than in the permanently flooded cores; and (iii) lower mitotic index values in *Allium cepa* cells exposed to water samples from the induced-to-drought cores during the rewetting period, leading to up to 22% less cell replication when compared to cells exposed to water samples from the permanently flooded cores. Understanding the effects of exposing aquatic sediments to the atmosphere and its subsequent flooding is a challenge, especially due to the wide variation in sediment quality. Therefore, it is critical to stimulate further efforts into this subject in order to comprehend the extent of these events in aquatic biogeochemical cycles, given that upcoming projections related to severe droughts appear to be an unavoidable fact.

## Supporting information

S1 File (XLSX)
